# Supplementary figures and images for: A novel method for quantitation of AAV genome integrity using duplex digital PCR
Source: PLoS One. 2023 Dec 14;18(12):e0293277. doi: 10.1371/journal.pone.0293277 (PMC10721069; doi:10.1371/journal.pone.0293277)

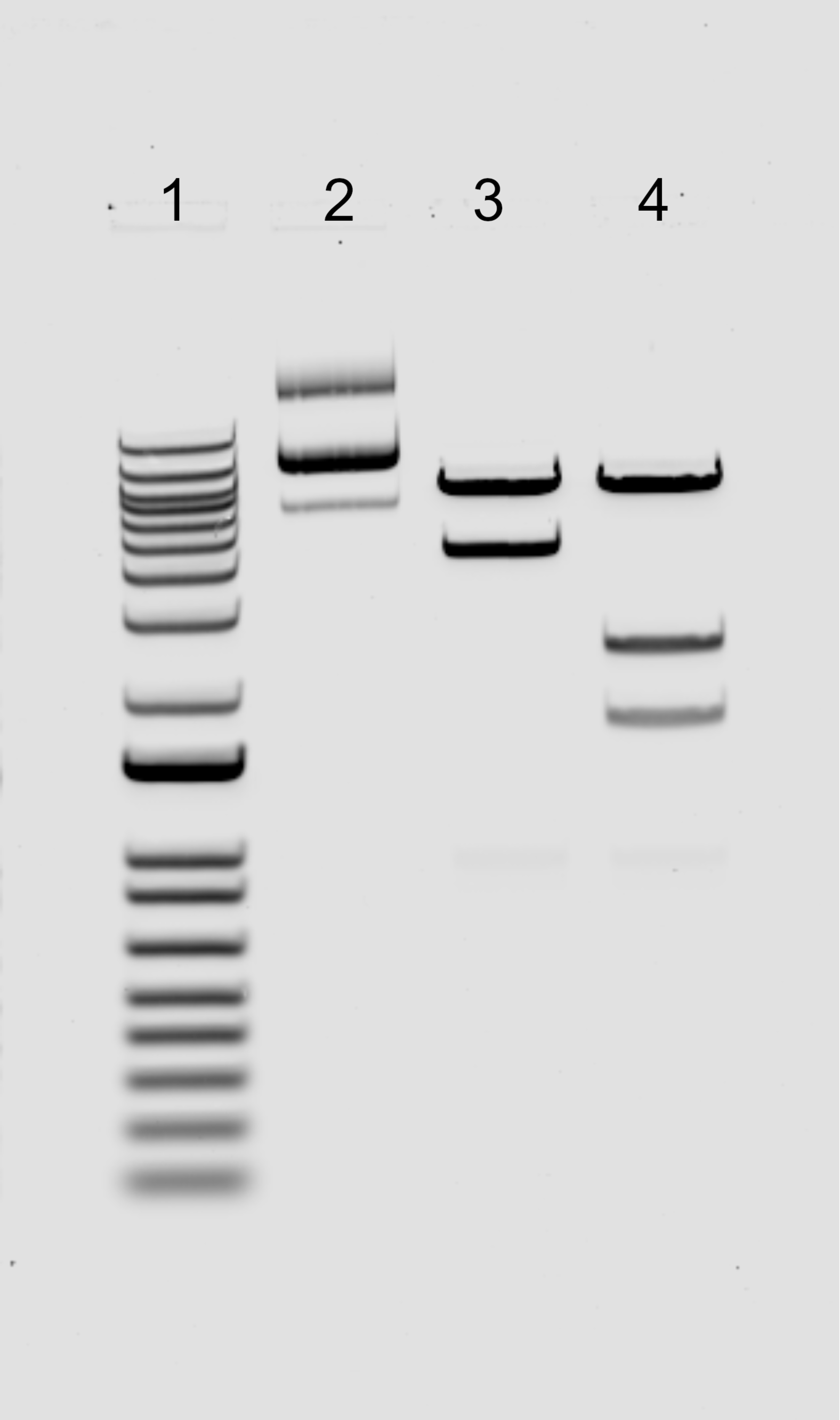

Supplement: S1 Fig — 1.5 μg of pAAV was digested with either MfeI alone, or with MfeI and NheI together for 1 hour at 37°C. Samples were run on a 1% agarose gel and visualized with SYBR Safe (Invitrogen) on an Odessey M imaging system (Licor). From left to right: Lane 1: Thermo 1KB ladder (10787018); Lane 2: undigested pAAV; Lane 3: pAAV MfeI digest; Lane 4: pAAV Mfe, NheI double-digest. Digested bands were of expected sizes (Lane 3: 7284, 4266; Lane 4: 7284, 2464, 1802). (TIF) [file pone.0293277.s001.tif]
