# Supplementary material for: A novel method for quantitation of AAV genome integrity using duplex digital PCR
Source: PLoS One. 2023 Dec 14;18(12):e0293277. doi: 10.1371/journal.pone.0293277 (PMC10721069; doi:10.1371/journal.pone.0293277)
Supplement: S1 Table — (PDF) [file pone.0293277.s004.pdf]

S1 Table. Linear fit information for linear models plotted in Figure 3D.

		Coefficient	Standard error	p-value
Linkage(avg) (pseudo R2 =0.972)	Intercept	14.3	3.89	0.003
	Slope	0.899	0.67	<0.001
Linkage(comp) (pseudo R2 =0.970)	Intercept	17.3	3.98	0.001
	Slope	0.877	0.07	<0.001
Poisson-multinomial (pseudo R2 1.000)	Intercept	-0.156	0.12	0.23
	Slope	0.968	0.002	<0.001
