# Supplementary material for: A novel method for quantitation of AAV genome integrity using duplex digital PCR
Source: PLoS One. 2023 Dec 14;18(12):e0293277. doi: 10.1371/journal.pone.0293277 (PMC10721069; doi:10.1371/journal.pone.0293277)
Supplement: S2 Table — (PDF) [file pone.0293277.s005.pdf]

S2 Table. Linear fit information for linear models plotted in Figure 6.

		Coefficient	Standard error	p-value
Linkage(avg) (pseudo R2 =0.989)	Intercept	8.31	2.48	0.002
	Slope	1.05	0.05	<0.001
Poisson-multinomial (pseudo R2 0.996)	Intercept	-1.16	0.59	0.058
	Slope	1.01	0.01	<0.001
