# Supplementary material for: A novel method for quantitation of AAV genome integrity using duplex digital PCR
Source: PLoS One. 2023 Dec 14;18(12):e0293277. doi: 10.1371/journal.pone.0293277 (PMC10721069; doi:10.1371/journal.pone.0293277)
Supplement: Attachment — Submitted filename: Response to Reviewers.docx [file pone.0293277.s007.docx]

**Response to Reviewers**

We thank the reviewers for their insights and suggestions. We feel that they greatly improved the clarity of the manuscript and added context to how the new methodology relates to orthogonal methods for AAV capsid and genome characterization. Please find our responses to each point interspersed with the reviewers’ comments (italicized) below:

***Response to reviewer #1:***

*Line 35. A colleague commented to me that long read methods including NGS methods are inferior in precision to dPCR when quantitating amounts of species. Better precision is a technical advantage which might recommend dPCR even if the practical reasons given relating to cost, effort, data analysis and amount of material. My second hand opinion is that even if NGS were improved to be faster, data analysis automated by better software, and miniaturized to use less sample, that primer based methods will still be preferred for higher precision. I suggest that you add a sentence or two based on your experience of NGS methods. First, a statement that NGS supports the method development of dPCR primers. And then a statement of the technical reasons why primer based dPCR can achieve higher precision than NGS methods.*

We appreciate Reviewer 1’s point about the limitations of the precision of NGS. We clarified our statement on the limitations of NGS in the introduction (lines 44-47). We also provided statements around the benefits of the accuracy/precision/sensitivity of duplex dPCR relative to NGS to the discussion section (lines 366-383).

*Line 320 Early discussions about electron microscopy use in the field gave me the opinion that Cryo EM worked much better than TEM but the industry tended to use TEM only because of cost despite its technical inferiority. The study in a paper by Werle et al that compared different methods on an unusual AAV sample with very low or missing partially empty capsids showed that even with no partially empty capsids present, TEM has precision issues. (Werle AK, Powers TW, Zobel JF, Wappelhorst CN, Jarrold MF, Lyktey NA, Sloan CDK, Wolf AJ, Adams-Hall S, Baldus P, Runnels HA. Comparison of analytical techniques to quantitate the capsid content of adeno-associated viral vectors. Mol Ther Methods Clin Dev. 2021 Sep 1;23:254-262. doi: 10.1016/j.omtm.2021.08.009. PMID: 34703846; PMCID: PMC8505359.) I suggest adding the Werle reference here and emphasizing that even in the simplest systems, TEM and other orthogonal methods have deficiencies in precision and dynamic range when quantitating percent intact genomes of AAV samples. (For instance, the EM they used always sees some empty or some full capsids even when the tested sample was monodisperse. Image recognition software is a bit imprecise.) The Werle paper also re-enforces the idea that all methods have technical and data analysis limitations that often require orthogonal information to correct. Too often instrument manufacturers distort the science by pressing the opinion that the “customer” will implement only one assay and using pecuniary pseudo-truths try to sell only one instrument.*

We agree with the reviewer’s suggestion to further emphasize the distinction between capsid characterization methods and genome characterization methods. We added a statement to the introduction to highlight the gap in capsid methodologies for characterizing genomes (lines 38-47). We have also added to the discussion as suggested, to now emphasize the value and necessity of employing orthogonal methods for characterizing capsid content and added several references, including the suggested reference Werle et al 2021 (lines 366-383).

*I commend the authors on using theoretical considerations, on very well characterized plasmid derived samples, and on AAV samples controlling the temperature stress as a parameter. This was an excellent experimental plan. The scheme to present diagrams and figures to visualize a mainly mathematical point, particularly the use of the %recovery parameter, was excellent.*

We thank the reviewer for these kind words regarding the experimental plan and presentation of the data.

***Response to Reviewer #2:***

*In this manuscript, Lauren Tereshko and collaborators have used multiplex droplet digital PCR to assess rAAV genome integrity. The originality of the manuscript resides in the comparison of different calculation formulas to analyze genome integrity ddPCR data. Interestingly, they have proposed a new and Poisson-multinomial model that improves the accuracy and quantifiable range of duplex ddPCR assays to determine rAAV genome integrity.*

*Only minor comments should be addressed:*

*(lines 30-34) Add citations for rAAV heterogeneity, residual DNA and purification process statements.*

We added several citations for rAAV heterogeneity and process impurities to the introduction (lines 36-38).

*(lines 58-59) Clarify the sentence “We compare the accuracy … genomes of 0-100% across a range of concentrations”. I assume 0-100% in length?*

We removed the 0-100% statement as it added confusion. The range of 0-100% simulated integrities corresponds to ranges in the percentage of the presence of both genetic termini from the population of template DNA molecules present. We added details regarding the preparation of mock integrity samples throughout the manuscript (see in particular lines 85-94). We hope these edits clarify that duplex dPCR reactions serve as a proxy for genome integrity by measuring the binary presence or absence of each genetic terminus and therefore do not directly measure genome length.

*Material and Methods must be fleshed out. Please provide primers and probe sequences and PCR cycles conditions. How was ITR restriction digestion performed? Give more details about NGS (library preparation, injected quantity, bioinformatics analysis)*

We have added significantly to the Materials and Methods section and now provide details for: DNA material identity, primer/probe sequences, thermocycling program, ITR digestion, NGS library preparation, sequencing, and analytical pipeline. We have added references to this section as needed.

*How genome fragments were generated, verified and quantified? Restriction enzyme sites can be illustrated on Fig1. Add an agarose gel with plasmid fragments as supplemental figure.*

Figure 1 was updated for clarity and includes the restriction sites. We have added a supplemental figure (S1 Fig) confirming the digestion of pAAV with MfeI alone and MfeI/NheI together. The image of the gel shows that the digested plasmid has the expected banding pattern for “intact” and “fragmented” genomes.

*Double stranded DNA is not degraded by 95°C heat* *(doi: 10.1089/dna.2013.2056). Is there another interpretation of your data? Heat effect on AAV single-stranded DNA? Fragments reannealing? A verification of AAV DNA degradation by heat using an orthogonal method (microfluidic capillary electrophoresis…) may be interesting.*

We appreciate Reviewer 2’s suggestion to verify the effects of heat on AAV DNA. In contrast to Karni et al 2013 (doi: 10.1089/dna.2013.2056), which tested the effects of heat on plasmid DNA, we have used linear dsDNA and ssDNA which have been shown to be less stable than circular DNA (Marguet and Forterre 1994 (https://doi.org/10.1093/nar/22.9.1681), Lindahl and Nyberg 1972 (https://doi.org/10.1021/bi00769a018)). Although Karni et al 2013 did not see significant degradation of plasmid DNA at 95°C, this was tested only with dry DNA for 5 minutes. In their discussion section, the authors state *“In an aqueous solution, it is not possible to determine the degradation temperature using our method, since applying pressure on the DNA solution causes the DNA to be more sensitive to heat, and therefore the DNA degrades already above 90°C.”* In addition to the two mentioned above, multiple other peer-reviewed publications have previously reported thermal degradation of dsDNA and ssDNA in aqueous solution. Our reference section has been updated to expand on these citations (see references 22, 25-31).

To address Reviewer 2’s suggestion, we have performed an alternative experiment. Unfortunately, we do not have enough of the ssDNA AAV material that was used in Figure 7 to visualize the degradation by electrophoresis. We instead linearized AAV vector plasmid DNA by restriction digest with BamHI, performed heat stress, and resolved the samples by agarose gel electrophoresis. Below are the results of incubating 500 ng samples of linearized plasmid DNA for 0, 5, 15 or 30 minutes at 95°C. The control lane (0 minutes at 95°C) shows the majority of DNA is of the expected length (~12 KB). Upon heat treatment, the band gradually becomes smaller. At 15 and 30 minutes at 95°C, smearing below the band can be seen at increasingly small sizes.

From left to right: Lane 1: Thermo 1KB ladder (10787018); Lanes 2-5: 0-, 5-, 15- and 30-minute incubation at 95°C.

While the data here demonstrate the thermal instability of linearized plasmid DNA, this does not directly correlate to the manuscript as Fig 7 uses AAV material; we therefore did not include this as a supplemental figure. However, we believe these data combined with our results from Fig 7, and the additional cited publications support our conclusion that rAAV DNA degrades into smaller fragments upon heat treatment thereby reducing the number of intact templates. Although re-annealing of ssDNA fragments could potentially occur, this alone would not affect measured genome integrities, as it would not change the ratio of the number of 5’ and 3’ targets that are present during PCR amplification cycles. Furthermore, we do not see changes in the concentration of ddPCR targets in the raw data across incubation times, which would be expected to decrease if annealing occurred.
